# Energy Efficient Cluster Based Scheduling Scheme for Wireless Sensor Networks

**DOI:** 10.1155/2015/185198

**Published:** 2015-10-01

**Authors:** E. Srie Vidhya Janani, P. Ganesh Kumar

**Affiliations:** ^1^Department of Computer Science and Engineering, Anna University Regional Office Madurai, Madurai 625 007, India; ^2^Department of Information Technology, K.L.N. College of Engineering, Pottapalayam 630 611, India

## Abstract

The energy utilization of sensor nodes in large scale wireless sensor network points out the crucial need for scalable and energy efficient clustering protocols. Since sensor nodes usually operate on batteries, the maximum utility of network is greatly dependent on ideal usage of energy leftover in these sensor nodes. In this paper, we propose an Energy Efficient Cluster Based Scheduling Scheme for wireless sensor networks that balances the sensor network lifetime and energy efficiency. In the first phase of our proposed scheme, cluster topology is discovered and cluster head is chosen based on remaining energy level. The cluster head monitors the network energy threshold value to identify the energy drain rate of all its cluster members. In the second phase, scheduling algorithm is presented to allocate time slots to cluster member data packets. Here congestion occurrence is totally avoided. In the third phase, energy consumption model is proposed to maintain maximum residual energy level across the network. Moreover, we also propose a new packet format which is given to all cluster member nodes. The simulation results prove that the proposed scheme greatly contributes to maximum network lifetime, high energy, reduced overhead, and maximum delivery ratio.

## 1. Introduction

*Wireless Sensor Networks.* A wireless sensor vetwork (WSN) comprises a large number of nodes either deterministically or deployed in a random manner, for instance, from an aircraft, to observe the environment. Sensor nodes communicate with each other by multihopping, that is, by using other sensor nodes in the network as relay nodes. In most applications, all the sensor nodes are required to transmit their data to a special node termed base station or sink which links the sensor network to the end-user, for instance to an airplane crossing the network area. Sink is assumed to have enormous energy resources, complicated processing and extreme transmission range capabilities together with adequate memory. One of the most important issues regarding the design of sensor networks is power consumption since these networks consist of sensor nodes densely deployed in hazardous and remote areas where the replacement of batteries is impossible. The major portion of energy is consumed during data transmission and reception. All these generic network operations together with the underlying computational operations also contribute to the energy utilization.

*Need for Scheduling in WSNs.* The WSNs comprise a collection of sensor nodes. Every one of these has an implanted processor, a radio, and one or more sensors. These nodes operate together in the area being observed and gather physical quantities of the environment, such as temperature or humidity. Information sensed by these sensor nodes can be utilized by different high level applications such as habitat monitoring, surveillance systems, and across systems monitoring various natural phenomena. Sensor nodes have restricted battery life. Most applications rely on the sensors deployed in hard-to-reach areas. In such scenarios, it is unrealistic for any kind of manual intervention to replace battery. The probability of these dispensable sensor nodes to stay alive within the network is based on their energy depletion levels. Hence the sensor network nodes are expected to manage themselves on their energy constrained battery resources. In the absence of optimal power management, the sensor nodes are believed to have a minimum lifetime.

Inherently, grouping sensor nodes into clustershas been widely adopted to achieve high energy efficiency and in turn to prolong network lifetime in large scale WSN environments. The proposed Energy Efficient Cluster based Scheduling Scheme (EECSS) implies cluster based organization of the sensor nodes in order that cluster head (CH) selection considering the residual energy parameter is made possible, thus leading to significant energy savings. The network structure allows each cluster to have regular sensor nodes as members and a cluster leader, which is also called the CH and quiet naturally performs the special scheduling tasks across the clustered sensor networks. CH nodes perform data aggregation and transmit them to the base station; hence they are expected to expose higher energy drain rates due to their excessive transmissions to remote locations at farthest proximities. The EECSS attempts to balance the energy consumption among all the network nodes, by periodically reelecting new CHs over time in each cluster across the network. Also the CH monitors the cluster members for their drain rates and based on it they are scheduled for each round of communication. The scheduling algorithm presented also allocates different time slots for the cluster members to send their energy information and actual data information to their corresponding CHs.

The need for clustering in WSNs is motivated in Section 1 and a briefing on the sensor network pattern is given. The state of the art in corresponding cluster based scheduling approaches for large scale WSN environment is presented in Section 2. Also the existing design challenges are explored. In the main body of the paper, that is, Section 3, the implementation methodologies for the proposed EECSS are discussed. The analysis of the experimental settings and results are discussed in Section 4. Finally the paper concludes with Section 5 stating various general comparisons between the proposed and existing methodologies.

## 2. Related Work

The Long-Lifetime and Low-Latency Data Aggregation Scheduling (L4DAS) algorithm [1] is proposed to build a degree-bounded least height spanning tree as an aggregation tree which provides an extended network lifetime. A maximum impedance priority scheduling algorithm is introduced to organize the transmission of nodes in such a way the latency is minimized considerably.

With reference to [2], a novel distributed Power Scheduling (PS) algorithm is presented for stationary continuous monitoring sensor networks. This scheme takes advantage of the time scale distinction between sensor network reconfiguration periods and data transmitting periods. The approach is distributed and works in a well-blended cooperation with well-defined sensor network routing and MAC layer protocols. However the approach hardly fits into any one layer, as it requires the joint effort of both the routing and MAC layers.

An Energy Efficient Distributed Schedule-Based (EEDS) protocol [3] is introduced for applications with periodic data traffic. The EEDS insists that the time frame be composed of rounds. Each round comprises three phases. In the first phase, an energy-aware tree is built, and then a TDMA schedule is constructed in a distributed fashion in the second phase. In accordance to the built schedule, the nodes are subject to data transmission in the final phase. At the mere initiation of each round, the tree is rebuilt and a fresh TDMA schedule is constructed.

The Packet Scheduling Algorithm (PSA) [4] is employed to lessen the packet congestion in MAC layer that ultimately reduces the overall packet collision in the network system. The algorithm allows scheduling ofall packets from application layer and network layer to reduce network congestion which in turn prevents packet collision across data link layer. The PSA implementation proves to minimize packet collision with an exponential increase in throughput as a by-product.

The Deadline Aware Energy Efficient Query Scheduling (DAEEQS) algorithm [5] is identified to be suitable for location-based queries in WSNs with mobile sinks. This scheduling algorithm aims to escalate the rate of successful queries and to curtail the energy expenditure of sensors with multihop communication that exploits deterministic sink mobility. For this reason, prior to query submission, the scheduling algorithm makes choice of the release and collects sensors such that two important performance criteria are met: the energy requirement for data forwarding is minimal and the response time meets the deadline specified.

The Mobile Element Scheduling (MES) cited in [6] outlines a scheduling problem where a mobile element needs to visit the sensor nodes so that none of their buffers overflow. It is proved to be NP- complete. It concludes with significant heuristics that prove that the Minimum Weighted Sum First algorithm performs extremely well and is computationally inexpensive. In addition it was formulated to adapt node failures.

The Efficient Scheduling for the Mobile Sink in Wireless Sensor Networks with Delay Constraint (ESWC) states unified framework [7] to analyze the sink mobility problem in WSNs with delay constraint. A mathematical formulation that jointly considers the issues of sink scheduling, data routing, bounded delay, and so forth, is depicted. A set of subproblems and optimal solutions are discussed. Finally the algorithm attempts to generalize the solutions and proposes a polynomial-time optimal approach for the origin problem.

An energy efficient scheduling technique [8] that addresses WSN environment in terms of lifetime maximization and delay is employed. In this paper, two optimization problems were focused. Firstly, when the wake-up rates of the sensor nodes are given, a distributed algorithm that minimizes the expected event-reporting delay from all sensor nodes to the sink is developed. Secondly, exploiting a specific definition of the network lifetime, lifetime-maximization problem is handled to optimally control the sleep-wake scheduling and the any-cast scheduling policies in order to maximize the network lifetime subject to an upper limit on the expected end-to-end delay.

In connection to the High Energy First (HEF) algorithm [9], the issue of predictability is addressed for WSNs of real-time interests. The HEF algorithm proves to be an optimal CH selection algorithm that maximizes a hard *N*-of-*N* lifetime. Also the paper concludes with theoretical bounds on the feasibility test for hard network lifetime with respect to the HEF.

The Balanced-Energy Scheduling (BS) scheme [9] is introduced in cluster based sensor networks. The aim of BS scheme is to evenly distribute the energy load of the sensing and communication tasks among all the nodes in the cluster, thereby extending the time until the cluster can no longer provide adequate sensing coverage. Two related sleep scheduling schemes, the Distance-Based Scheduling (DS) scheme and the Randomized Scheduling (RS) scheme, are also studied in terms of the coefficient of variation of their energy consumption.

The wireless sensor network lifetime is improved through Power Aware (PA) organization [10]. The problem of energy efficiency in wireless sensor applications is addressed for surveillance of a set of targets with known locations. It is considered that a large number of sensors are dispersed randomly at closest proximities to a set of objectives and the monitored information is sent to a central processing node. The principle behind that is that every target must be monitored at all times at least by one sensor and every sensor is able to monitor all targets within its operational range. The method proposed for extending the sensor network lifetime is to divide the set of sensors into disjoint sets such that every set completely covers all targets. As all targets are monitored by every sensor set; the goal of this approach is to determine a maximum number of disjoint sets, so that the time interval between two activations for any sensor is longer.

The Dynamic Conflict-Free Query Scheduling (DCQS) [11] presents a novel transmission scheduling technique specially designed for query services in large scale WSNs. The planner cuts down query latency by constructing transmission plans based on the precedence constraints with in-network aggregation. The scheduler enhances throughput by overlapping the transmissions of multiple query instances concurrently while enforcing a conflict-free schedule. Also it attempts to achieve a maximum query rate.

The local wake-up scheduling (LWS) [12] is an ant colony based scheduling method to prolong the network lifetime with full coverage impulsion. In this method, the artificial ants search solutions in a two-phased manner. The first phase finds a set of sensors that satisfy full coverage constraint while the second phase finds the successors of sensors which run out of energy. In addition, graphs are designed to guide the artificial ants to search partial solutions in the two phases.

An Energy Efficient Adaptive Sensor Scheduling (EEASS) [13] accounts for target monitoring algorithm in WSNs. A motion monitoring algorithm is devised based on energy balance in local monitoring region of WSNs. An improved particle filter algorithm for degeneracy is applied to localize the target. Due to the redundant information collected from sensor nodes in the cluster, a sensor selection method is felt essential. The problem of sensor selection is exchanged into energy optimization subject to the decision function and the joint detection probability. Sensor nodes with higher decision values are selected under the condition of the joint detection probability and superior performances (i.e., tracking accuracy, residual energy of a sensor node, accumulated energy consumption, and lifetime of WSNs) for target tracking.

As indicated in [14], the scheduling algorithm introduced for Wireless Multimedia Sensor Networks (WMSNs) divides the frame sent from the cluster head to the base station into slots and gives a certain percentage of these slots to each node. These percentages are calculated taking into account the node priority and the strength of the flow from that node into the CH. The percentage is subjected to recalculation each time the CH receives a new query with new nodes involved. In WMSNs the multimedia streaming might cover eventually long periods of monitoring; the queries will be separated by certain periods of time and thus the recalculation does not impose a burden on the scheduler.

The Energy Efficient Routing Scheme for Sensor Networks Using Connected Dominating Set (CDS) [15] highlights a power aware routing approach with two phases. The first phase discusses the sparse topology over the visibility graph of the network in a localized manner. The second phase computes the data gathering tree over the edges of the computed sparse topology. The overall routing hierarchy inherits the flooding technique.

The survey concludes that most attention has been focused on the issues relevant to small sensor networks. However, the significance of the large scale WSNs in interaction with the outside world is not considered. Also the techniques addressed are found to be application specific. Therefore, cooperation among sensor networks creating groups of different sensor networks that accounts to a large scale WSN could be an area worth looking into.

## 3. Implementation of Proposed Scheme

An Energy Efficient Cluster based Scheduling Scheme (EECSS) that emphasizes enhancing network lifetime and curtail energy consumption using cluster based scheduling approach is recommended. Likewise, there are several related existing works highlighting the energy and lifetime parameters of individual sensor nodes. In contrast, our scheme enunciates the lifetime of cluster groups based on prioritised CH selection and total energy consumption of cluster nodes. In the proposed scheduling algorithm, time slots are assigned to CHs and cluster members. The sensor nodes are continuously monitored during listening, sleeping, and transmission phases. Moreover, the cluster topology is discovered to increase network lifetime and attain maximum residual energy across the entire network. In cluster model, the CH is being chosen for managing the connectivity of all the cluster member nodes.

### 3.1. High Energy Cluster Topology

**Figure 1:**
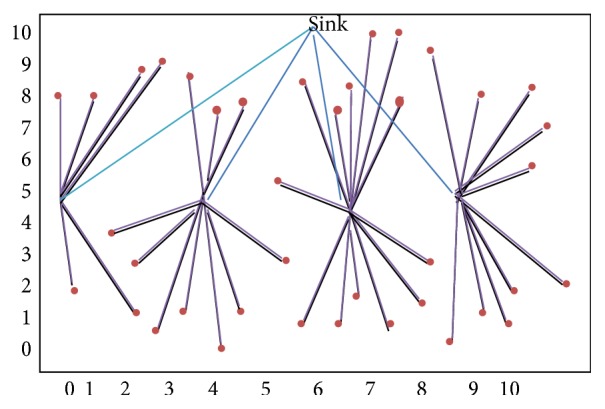
Cluster topology.

In the proposed high energy cluster topology, network is organized as clusters, where each cluster member is associated with CH. Here, no base station is implemented. Cluster members and CHs communicate without any centralized infrastructure. Each cluster member node transmits the data via CH. The CH is chosen based on remaining energy level. The sensor nodes with maximum remaining energy levels are employed as CHs. The implementation of cluster topology relies on time slot based scheduling algorithm. In general, CHs are nodes that expose highest energy consumption due to their continuous data transmissions to remote base stations. Hence the probability for CHs to run out of energy is comparatively very high. To overcome this, the cluster topology employs new CHs based on the residual energy parameter. This paves the way for the energy consumption to be balanced across the entire network which in turn increases the network lifetime. In addition, there is a tradeoff between energy consumption and distance between CH and cluster members. With cluster topology, each cluster has at least one CH. Hence sensor networks that comprise multiple clusters have multiple CHs, which consume more energy. Our approach attempts to balance the energy consumption by allowing the sensor node with highest residual energy to act as CH at each round of communication. Hence the cluster topology proposed ensures maximum number of nodes with high residual energy as shown in Figure 1.

*Cluster Topology Discovery Procedure for CH Selection.* In a cluster group, each cluster member sensor node generates a random energy probability (*E*_*t*_) at the beginning of a cluster discovery and computes the threshold value (*τ*(*k*)) with the use of equation:

(1) τk=Et1−Etm·mod⁡⁡1/Et·EremEmax⁡k∈N0otherwise,

where *N* is the set of cluster groups, *E*_rem_ is the remaining battery energy, *E*_max⁡_ is the maximum battery energy of the sensor node, *E*_*t*_ is the desired percentage of CHs, and *m* is the current round number. In case of *E* < *E*_*t*_, the node is selected as a CH.

A chosen CH broadcasts data packets to neighbouring cluster member nodes. The cluster member nodes collect the data messages for a given time interval and then send a “LINK-REQ” message to the closest CH. On receiving the “LINK-REQ” message the CH builds a cluster member routing table list and a time slot based schedule given by Time Division Multiple Access (TDMA). The time slots generated are broadcasted to the neighbouring cluster member nodes. The time slots convey the time instants for the cluster member nodes to send their data and energy information to their corresponding CHs. In turn the member nodes receiving the time slots restore the same for data transfers. Once cluster selection process is over, each cluster member sends data and its remaining energy information to the corresponding CH as per the given time slot schedule. Consecutively, the CH maintains remaining energy information of cluster member nodes.

In cluster topology presetup phase, a CH sends out the maximum residual energy value of all its cluster member nodes to another CH before last round of transmission expires. In this manner, each CH collects the remaining energy values from all CHs and attempts to find the maximum remaining energy value (*E*_max⁡_) of the network. Ultimately, the *E*_max⁡_ computed is communicated to all other CHs. The CH broadcasts Emax to cluster member sensor nodes. Each node saves the value of *E*_max⁡_ for the subsequent computation of  *τ*(*k*) and the current round is terminated.

### 3.2. Scheduling Algorithm

The scheduling algorithm proposed identifies the sensor nodes based on their active, idle, and sleep modes. The time ON and time OFF factors are calculated for nodes that are either in sleep or in active mode in order to allocate time slots to all cluster member nodes. The waiting time for cluster member sensor nodes is calculated based on their status of listening, sleeping, and activation stages. In addition, an assumption is made that the sensor nodes considered are mobile in nature and the clusters get reorganised as a function of time.

**Step 1 (compute ON (*t*_ON_) and OFF time (*t*_OFF_)).**

The *t*_ON_ represents the duration of time for which a sensor node is on:

(2) $$\begin{array}{c} t_{\text{ON}}=\frac{f-r}{v_{\max}}, \end{array}$$

where  *f* − *r* is the minimum time for the sensor node to get into coverage and *V*_max⁡_ is the maximum sensing range of the node.

The *t*_OFF_ represents the duration of time for which a sensor node is off:

(3) $$\begin{array}{c} t_{\text{OFF}}=\min\left\{\;\frac{f+r}{v_{\min}},t_{\exp}\;\right\}, \end{array}$$

where *f* + *r* is the maximum time for the sensor node to get into coverage and *V*_max⁡_ is the minimum sensing range of the node.

**Step 2 .**

Each cluster has its own identifier and each cluster member node is identified by a unique codeword within the cluster.

**Step 3 .**

Update current remaining energy *E*_rems_.

**Step 4 .**

Collect information and construct the set of cluster groups.

**Step 5 .**

Compute the waiting time *w*_ck_ and start the decision phase timer *t*_ON_. The waiting time for the cluster member sensor nodes is calculated based on their status of listening, sleeping, and activation:

(4) $$\begin{array}{c} w_{\text{ck}}=\left\{\begin{array}{cc} \frac{\eta }{k_{i}^{\chi }l{\left(e_{i},v_{i}\right)}^{\alpha }}\times W+z & e_{i}\geq e_{T}\\ W & \text{otherwise}, \end{array}\right. \end{array}$$

where *η*, *χ*, *α* are the constants, *e*_*T*_ is the threshold energy level, *z* is a random number between [0 · *t*_ON_], and *l*(*e*_*i*_, *v*_*i*_) is the function computing the lifetime of cluster groups in terms of current energy *e*_*i*_ and its sensing range *v*_*i*_.

**Step 6 .**

Status = LISTENING

**Step 7 .**

Precheck redundant neighbors, whether nodes are in sleep state. Send message to them and move them out of list *L* if found any.

**Step 8 .**

*n*_*i*_ = number of elements of list *L*

 while *t* ≤ *W* do
 Receive (CM_*i*_, MessageID)
 if MessageID == kACTIVATE (Activated message)
 then

**Step 9 .**

Update coverage level. Check if any sensor in list *L* is useless to CM_*i*_'s coverage.

 If yes, kATSLEEP (Nodes are in sleep mode) send message to that sensor
 else if MessageID == kATSLEEP then
 if *n*_*i*_ > 0 and status == LISTENING then

**Step 10 .**

Update *w*_ck_

 if (*t* ≤ *w*_ck_ and status == LISTENING) or *n*_*i*_ = = 0
 then
 if *n*_*i*_ = = 0 then
 Set the timer Ri for si waking up at next round
 else
 status = ACTIVE
 Set itself to be* Active*

**Step 11 .**

One-hop broadcast kACTIVATE message

### 3.3. Energy Consumption Model

The free space propagation model is utilised for modelling the energy consumption of a sensor node during transmission, reception, idle, and sensing states. The modelling takes into account the distance over which the sensor nodes communicate. Hence, the transmission energy of CH over a distance *d* is given as

(5) Etxn,dEtx-elecn+Etx-ampn,d=nEelec+nξfsd2d<d0nEelec+nξampd4d≥d0,

where *E*_elec_ is the transmitter circuitry dissipation per bit. Consider

(6) $$\begin{array}{c} E_{\text{rx}}\left(\;n\;\right)=E_{\text{rx-elec}}\left(\;n\;\right)=nE_{\text{elec}}. \end{array}$$

Our main goal is to minimize the network cost in terms of energy, to prolong the lifetime. The mathematical model is given as *Min*⁡(*E*_tot_), that is, the minimum total energy expenditure:

(7) $$\begin{array}{c} E_{\text{tot}}=E_{t}+E_{r}+E_{i}+E_{s}, \end{array}$$

where *E*_tot_ is total energy expenditure across the network, *E*_*t*_ is the energy consumed during transmission, *E*_*r*_ is the energy consumed during reception, *E*_*i*_ is the energy spent when the nodes are in idle state, and *E*_*s*_ is the energy spent for sensing. In the energy consumption model, *E*_*r*_, *E*_*i*_, and *E*_*s*_ are constant and *E*_*t*_ varies based on the distance covered during transmission.

### 3.4. Proposed Packet Format

In Table 1 the parameters Source Cluster ID and Destination Cluster ID uniquely identify the Source and Destination clusters for a particular round of communication. Scheduling Status indicates the status of the node to be either active, sleep, or listening. Network lifetime indicates how long a sensor node lives with optimum remaining energy level within the current cluster. Total Energy Level is the overall energy of the cluster group which is monitored by all CHs. Frame Check Sequence (FCS) is to identify duplications across the frames transmitted.

**Figure 2:**
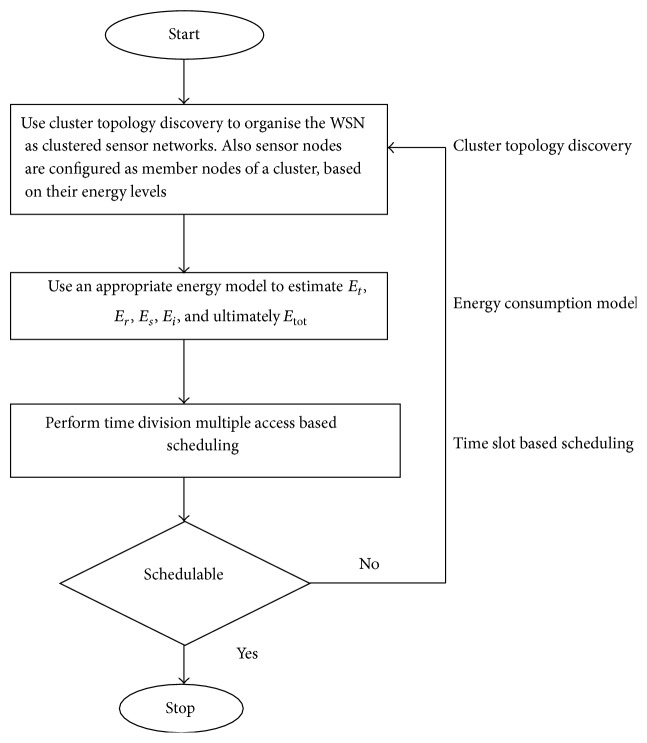
Flowchart of proposed scheme.

Figure 2 represents the workflow of the proposed system. Initially, the cluster topology discovery phase is primly set out to organise the WSN as a clustered sensor network. Here the sensor nodes are configured as member nodes of a cluster only based on their residual energy levels. Then, with the integration of the energy consumption model, the energy spent during various network tasks is estimated. Finally, the time slot based scheduling is performed in the basis of Time Division Multiple Access. The set of sensor nodes that are schedulable for a particular round of communication are identified.

## 4. Simulation

In this section, we demonstrate that the derived results above are consistent with simulation results. We use NS2.34 to conduct a performance study to compare the performance of EECSS with that of HEF and investigate the feasibility of EECSS. There are 200 sensor nodes, organized in a random topology and randomly deployed in a square region 1200∗1200 meters in size. The simulation is prone to a maximum of 60 seconds. The entire sensor nodes are considered to have a transmission range of 500 meters. The simulation parameters are listed in Table 2.

### 4.1. Performance Metrics

We evaluate the performance of the work proposed in terms of the following.

*End-to-End Delay.* The simulation averages the time taken for all surviving data packets to be transmitted across the network from sources to destinations.

*Packet Delivery Ratio.* It represents the ratio of packets that are successfully delivered to a destination compared to the number of packets that have been sent out by the sender. The simulation in turn generalises this successful delivery ratio across entire sources and destinations identified in the network.

*Throughput.* In terms of throughput the simulation attempts to average the overall rate of successful message delivery across all communication channels or communication links present in the network.

*Communication Overhead.* The total number of control packets that convey control information is normalised by the total number of actual data packets and hence a least communication overhead is experienced during the simulation.

*Energy Consumption.* The EECSS relates energy consumption to different states (transmit, receive, idle, and sense) of sensor nodes. With the high energy cluster topology, the simulation leads to uniform distribution of energy consumptionacross the network.

*Network Lifetime.* With higher emphasis on the efficient use of the critical network resources such as battery power, the EECSS prolongs the network lifetime in terms of energy retained in the individual sensor nodes and across clusters. The energy consumption model integrated allows our simulation to achieve maximum sensor nodes with optimal residual energy.

The simulation results are presented. Also a comparative analysis that proves our proposed EECSS to outperform one among the relevant existing High Energy First Clustering (HEF) algorithm [9] is discussed in the following sections.

The experimentations discussed in the following section demonstrate EECSS to surpass HEF by taking into account network lifetime when energy consumption is balanced across the network.

**Figure 3:**
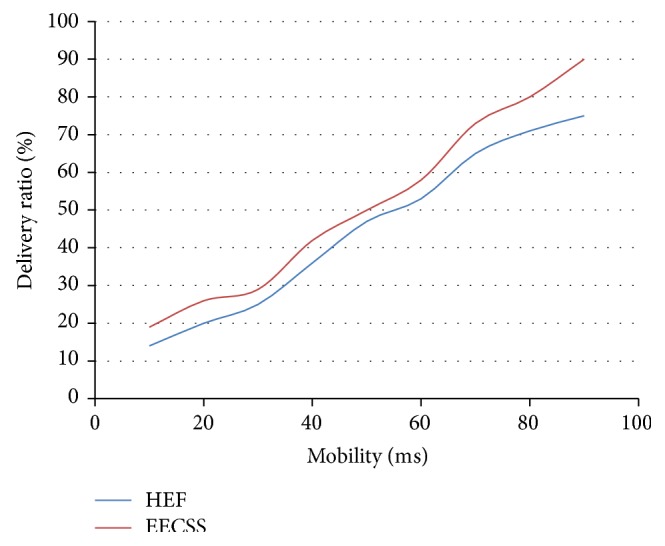
Number of nodes versus delivery ratio.

The comparison results between EECSS and HEF in terms of delivery ratio are presented in Figure 3, where the *Y*-axis represents the delivery ratio and the *X*-axis denotes the mobility. The delivery ratio is varied in terms of mobility. With EECSS an increase in the mobility of sensor nodes leads to a phenomenal increase in successful delivery ratio and it proves to be better than HEF. Also our simulation generalises this successful delivery ratio across all the sources and destinations identified within the network and proves that maximum number of packets are delivered at destinations.

**Figure 4:**
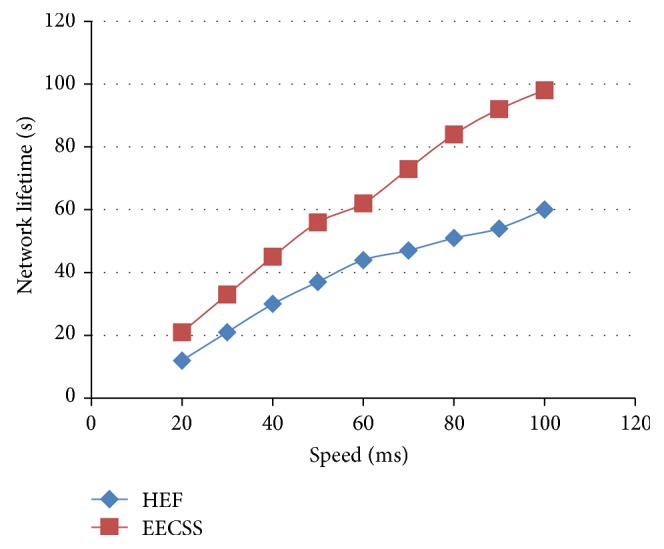
Speed versus network lifetime.

In Figure 4 the *X*-axis refers to the speed of the mobile sensor nodes and the *Y*-axis refers to the corresponding network lifetime. By varying the speed from 20 ms to 100 ms, EECSS proves to be better than HEF in terms of network lifetime because of its cluster topology and time based scheduling.

**Figure 5:**
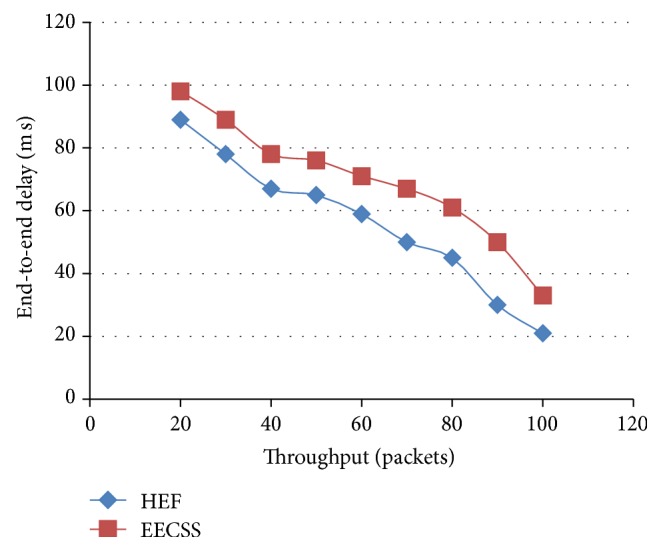
Throughput versus end to end delay.

The performance of EECSS in contrast to HEF in terms of end-to-end delay and Network Throughput is presented in Figure 5. The *Y*-axis represents the end-to-end delay and the *X*-axis represents the Network Throughput. The proposed EECSS shows a decrease in delay when throughput increases. Also it outperforms HEF in terms of latency. It shows a minimal end-to-end delay even when the sum of data traffic to the destinations is large. Hence EECSS is found to be suitable for large scale WSNs.

**Figure 6:**
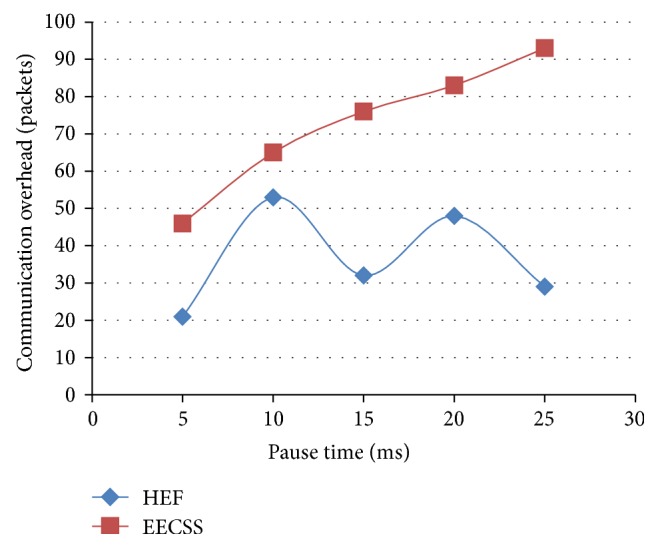
Pause time versus communication overhead.

In Figure 6 the *X*-axis refers to the pause time and the *Y*-axis represents the communication overhead. The simulation allows nodes to stop for a duration defined by the “*pause time*,” after which it again chooses a random destination and repeats the whole process. With EECSS the total number of packets carrying actual data outplays the total number of packets carrying control information. Hence EECSS shows a phenomenal decrease in communication overhead compared to HEF.

**Figure 7:**
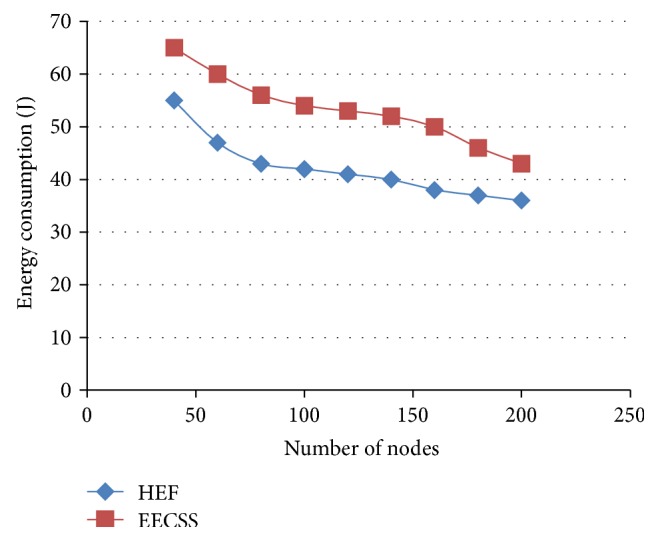
Number of nodes versus energy consumption.

The comparison results between EECSS and HEF in terms of energy consumption are presented in Figure 7, where the *Y*-axis represents the energy consumption and the *X*-axis denotes the number of nodes in the network. In EECSS, even if the network is scaled to a larger scale network supporting *N* number of nodes, energy consumption across the network decreases as the total number of network nodes increases. This is definitely achieved by the integration of high energy cluster topology and time based scheduling. The total energy consumption is balanced across the entire network which is not achieved in HEF.

## 5. Conclusion

In general, clustering in WSNs has been of high interest across research and industrial communities in the last decade. Throughout this work we have concentrated on the main characteristics of sensor networks such as network lifetime and energy consumption. As it was pointed out, EECSS paves way for grouping nodes into clusters, thus leading to energy efficient routing and scheduling has been proved as one among the most efficient approaches to support scalability in WSNs. Since it greatly reduces the total amount of communications as well as the energy spent, independent of the growth of WSN, it becomes highly suitable for real-time large scale WSNs. In contrast to the existing protocols that are in focus only towards the efficient utilization of critical battery resources which counts for the lifetime of the sensor nodes and ultimately lifetime of the sensor networks, our proposed work combines the need for fast convergence time and minimum energy consumption with regard to the cluster formation procedure. Moreover, nodes are assumed to make fast decisions, that is, to become CHs or not, based on their residual energies and not based on any random probabilities. The most critical feature of EECSS is the periodic reelection of CHs, based on residual energies among all the nodes of the network leading to uniform distribution of energy consumption. Habitually, it introduces a multi-level cluster hierarchy to preserve energy efficiency independent of the growth of the network in terms of number of sensors, area covered, and so forth, and it well suits real-time large scale WSNs. Finally, a few additional issues should be explored in future research. Some of these issues include the evolution of a comprehensive method for finding the optimal number of clusters in order to maximize the energy efficiency, the estimation of the optimal frequency of CH rotation/reelection to gain better energy distribution, the incorporation of several security aspects to enhance protection in hostile environments, and the further development of recovery protocols in case of CHs failure.

## Figures and Tables

**Table 1:** Proposed packet format.

Source Cluster ID	Destination Cluster ID	Scheduling status	Network lifetime	Total energy level	FCS
2	2	4	4	4	2

**Table 2:** Simulation parameters.

Parameters	Value
Number of nodes	200
Network size	1200 × 1200
Mac	802.11
Radio range	500 m
Simulation time	60 sec
Traffic source	Constant bit rate (CBR)
Packet size	512 bytes
Mobility model	Random way point

## References

[1] Chen Z., Yang G., Chen L., Wang J. (2012). An algorithm for data aggregation scheduling with long-lifetime and low-latency in wireless sensor networks. *International Journal of Future Generation Communication and Networking*.

[2] Sichitiu M. L. Cross-layer scheduling for power efficiency in wireless sensor networks.

[3] Al-Khdour T. A., Baroudi U. (2010). An energy-efficient distributed schedule based communication protocol for periodic wireless sensor networks. *The Arabian Journal for Science and Engineering*.

[4] Jandaeng C., Suntiamontut W., Elz N. (2011). PSA: the packet scheduling algorithm for wireless sensor networks. *International Journal on Applications of Graph Theory in Wireless Ad Hoc Networks and Sensor Networks*.

[5] Karakaya M. (2013). Deadline-aware energy-efficient query scheduling in wireless sensor networks with mobile sink. *The Scientific World Journal*.

[6] Somasundara A. A., Ramamoorthy A., Srivastava M. B. Mobile element scheduling for efficient data collection in wireless sensor networks with dynamic deadlines.

[7] Gu Y., Ji Y., Li J., Zhao B. (2013). ESWC: efficient scheduling for the mobile sink in wireless sensor networks with delay constraint. *IEEE Transactions on Parallel and Distributed Systems*.

[8] Lokhande P. M., Thakare A. P. (2013). An efficient scheduling technique for the improvement of WSN with network lifetime & delay constraint. *International Journal of Recent Technology and Engineering*.

[9] Cheng B.-C., Yeh H.-H., Hsu P.-H. (2011). Schedulability analysis for hard network lifetime wireless sensor networks with high energy first clustering. *IEEE Transactions on Reliability*.

[10] Cardei M., Du D.-Z. (2005). Improving wireless sensor network lifetime through power aware organization. *Wireless Networks*.

[11] Kannadas P., Daniel S. J. (2012). Energy efficient conflict free query scheduling for wireless sensor networks. *International Journal of Engineering and Advanced Technology*.

[12] Zhong J.-H., Zhang J. Energy-efficient local wake-up scheduling in wireless sensor networks.

[13] Zhang J., Wu C.-D., Zhang Y.-Z., Ji P. (2011). Energy-efficient adaptive dynamic sensor scheduling for target monitoring in wireless sensor networks. *ETRI Journal*.

[14] Watfa M. K., Shahla F. A. (2009). Energy efficient scheduling in WMSNs. *INFOCOMP Journal of Computer Science*.

[15] Sampoornam K. P., Rameshwaran K. (2011). Energy efficient routing scheme for sensor networks using connected dominating set. *International Journal of Computer Science Issues*.

